# Effect of Selenium on HLA-DR Expression of Thyrocytes

**DOI:** 10.1155/2012/374635

**Published:** 2012-02-02

**Authors:** Csaba Balázs, Viktória Kaczur

**Affiliations:** Department of Medicine and Endocrinology, Polyclinic of the Hospitaller Brother of St. John of God in Budapest, Budapest 1027, Hungary

## Abstract

Autoimmune thyroid diseases (ATDs) represent the most frequent forms of the organ-specific autoimmune thyroid disorders that result from interaction between genetic and environmental factors. Selenium has been shown to exert a beneficial effect on the autoimmune thyroiditis. In spite of therapeutical effect of selenium on autoimmunity, the mechanism of its action has not been revealed. *Objective*. To determine whether selenium in vitro thyrocytes cultures are able to influence the HLA-DR molecule expression of human thyrocytes and production of free oxygen radicals. *Method*. Thyrocytes were prepared from human thyroid gland and cultured in vitro in the presence of interferon-*γ* and sodium selenite. The expression of HLA-DR molecules induced by interferon-*γ* in the presence of sodium selenite of various concentration was measured by fluorescence-activated cell sorter. *Results*. Selenium has a dose-dependent inhibitory effect on the expression of HLA-DR molecules of thyrocytes induced by interferon-*γ*. This effect of selenium was in the inverse correlation with antioxidative capacity. *Conclusion*. Beneficial effect of selenium on autoimmune mechanism is a complex mechanism in which the inhibitory effect on HLA-DR molecule expression and antioxidative capacity are involved into therapy of autoimmune thyroiditis.

## 1. Introduction

Recently published clinical studies on possible effects of selenium (Se) in autoimmune thyroiditis evoked exciting discussion. The conflicting data were published on effect of Se; the one part of investigators provided evidences that Se intakes may be beneficial with respect to autoimmune diseases [1–7], the others were not able to show the significant effect of Se on autoimmune thyroiditis [8, 9]. Furthermore, the authors who published the beneficial effect of Se on the levels of autoantibodies, advised to use Se therapy for patients with autoimmune thyroiditis (AIT) [1, 4, 10]. Recently, we published our prospective placebo-controlled prospective study including 132 patients with autoimmune thyroiditis [4]. L-thyroxine substitution therapy was made in both groups and the level of TSH remained in the normal range. Se therapy was continued by L-seleno-methionine (per os 2 × 100 *μ*g/day) for one year. The level of Se in the untreated patients' sera was significantly lower than in treated patients and controls, and after three-month therapy with it, Se was normalized. The titer of antithyroid antibodies (mostly the anti-TPO) significantly decreased at the end of study. An inverse correlation was found between antioxidant capacity and level of anti-TPO antibodies. This observation suggests that Se deficiency by itself might be responsible for the precipitation of inflammatory process. Although the precise mechanisms of action and the possible targets of Se have not been clarified yet, the beneficial influence of Se can be explained by different points of views. Growing evidence supports that the selenium-containing enzymes and their antioxidant capacity somehow modify the autoimmune mechanism [11–15]. Previously, it was published that unlike in thyroids from healthy individuals thyroid epithelial cells from patients with AITD were able to express HLA class II antigen molecules similar to normally expressed on antigen presenting cells (APCs) such as macrophages and dendritic cells [16–19]. The aberrant expression of HLA Class II molecules on thyroid cells may initiate and perpetuate thyroid autoimmunity via direct autoantigen presentation. We removed the repeated references from the highlighted part. Please check similar cases throughout the paper. Previously we provided evidence for the role of HLA-DR expression on thyrocytes induced by interferon-*γ* (IFN-*γ*) and its modification by methimazole which has a significant anti-oxidative capacity [20]. It was assumed that the Se, like methimazole, can modify the expression of HLA-DR molecules on thyrocytes culture in the presence of Se; therefore, we made in vitro experiments using human thyrocytes cultures to study this hypothesis.

## 2. Materials and Methods

We cultured human thyrocytes and analyzed HLA-DR antigen expressions induced by IFN-*γ* in the various concentrations of sodium selenite (Sigma) in culture media by previously published method [16]. Briefly, thyroid epithelial cells were separated from surgical specimens. 4–6 × 10^6^  cells were obtained from 10 g of tissue with viability of >90% which was determined by trypan blue exclusion.  2 × 10^5^ cells were placed in each well of a 24-well Costar culture plate and cultured in minimum essential medium containing 15% fetal calf serum (FCS) with 0.2% sodium bicarbonate either alone (control wells) or in the presence of IFN-*γ* (Hoffmann-La Roche), and to other wells 10.0, 50.0, and 100 nmol/mL of sodium selenite (Sigma) were added. In most of experiments, thyrocytes were cultured for 3 days and then detached by 0.2% trypsin. HLA-DR expression was investigated initially (0 day) and on day 3 and 7 of culture. Cells were recovered in Ca^++^ and Mg^++^ free EGTA solution with rubber policeman. The detached cells were resuspended in RPMI containing 10% FCS, 10 mM HEPES (Sigma). For indirect immunofluorescence, cells were resuspended in 200 *μ*L RPMI (Sigma) containing 10% FCS, in 10 mM HEPES and were incubated for 30 min at 4°C with 5.0 *μ*L monoclonal anti-DR framework antibody (DAKO). The cells were washed twice and the pellet incubated for 30 min at 4°C with 1 : 100 dilution of FITC-conjugated rabbit anti-mouse immunoglobulin (Cooper Biomedical, Inc., Malvern, PA). After two washes, the cells were analyzed in fluorescence-activated cell sorter (FACS III, Beckton-Dickinson Co., Sunnyvale, CA). All experiments were made in triplicate. The total antioxidant status (TAS) was determined by Randox kit (Randox Laboratories Ltd, Crumlin, UK) [4, 12, 14].

### 2.1. Statistical Methods

The computerized program “Stat View” (Version 4.5, SAS Institute Corp., North Carolina, USA) was used for evaluating data. Descriptive statistics, Pearson's chi-square test, Fisher's exact test, and ANOVA analysis were performed. Values of *P* < 0.05 were considered as significant.

## 3. Results

**Figure 1:**
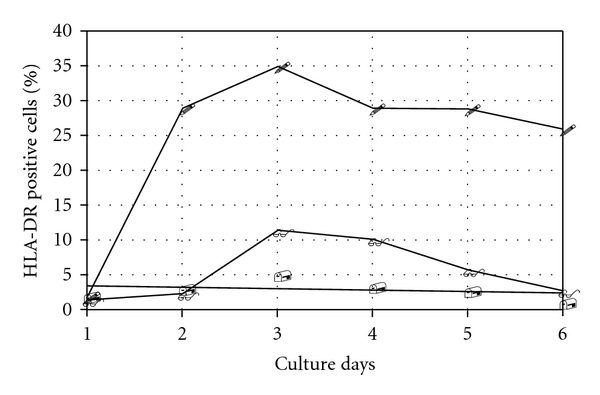
Time-dependent effect of sodium selenite (100 nM/mL) on IFN-*γ* (100 U/mL) induced HLA-DR expression. (i) Dots: HLA-DR expression without sodium selenite. (ii) Square: HLA-DR expression with sodium selenite (100 nM/mL). (iii) Filled square: HLA-DR expression with sodium selenite.

**Figure 2:**
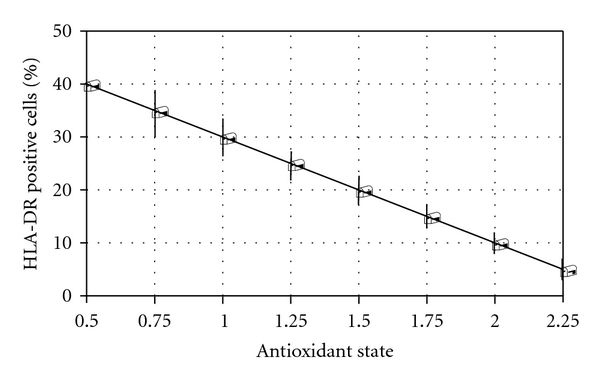
Study of connection between antioxidant status and expression of HLA-DR positive cells. Bars show the ±SD, *r* = − 0.72.

We found that IFN-*γ* (100 U/mL) was able to induce a significant stimulation of expression of HLA-DR molecules in thyrocytes (Table 1) (35.2 ± 15.2 versus 3.7 ± 2.4, *P* < 0.001). The peak of HLA-DR expression was at day three and then decreased abruptly. Therefore, we tested the expression of HLA-DR positive cells induced by IFN-*γ* at day three in absence and presence of Se of various concentrations. Se in two different concentrations (50 nM/mL and 100 nM/mL, resp.) significantly inhibited the expression of HLA-DR positive cells induced by IFN-*γ* (Table 1). If we added the Se to thyrocytes cultures after or before exposition of IFN-*γ*, there were not observed significant changes in HLA-DR expression. Time-dependent effect of sodium selenite (100 nM/mL) on IFN-*γ*-induced HLA-DR expression(100 U/mL) was on Figure 1. Then we studied the possible connection with HLA-DR expression on thyrocytes and antioxidant capacity of Se and found an inverse correlation between antioxidant status and expression of HLA-DR positive cells (*r* = −0.72, *P* < 0.01) (Figure 2).

## 4. Discussion

The trace element of Se plays an important role in the thyroid gland under physiological conditions and in diseases as well. Se supplementation decreased inflammatory activity in patients with autoimmune thyroiditis, and the reduction of titres of anti-TPO antibodies was correlated with serum levels of Se [2, 4, 6, 7]. Convincing observation was published for beneficial effect of Se in a patent with autoimmune thyroiditis when a marked decrease in thyroid 18FDG uptake after Se supplementation was found [21]. In spite of great efforts, the precise mechanism of Se has not yet been clarified. The role of antioxidant property of Se was published to be involved into beneficial effect in autoimmune thyroiditis [12–15]. Previously, we found that methimazole proved to have antioxidant capacity decreased the expression of HLA-DR molecules on the surface of thyrocytes [20]. Our experiments confirmed that the Se has a significant radical scavenging effect and the decrease the expression of HLA-DR molecules induced by IFN-*γ* was in an inverse correlation with antioxidative capacity of thyrocytes supernatant. The exogenous factors including iodine and oxidative stress have been published to be precipitating factors in genetically susceptible individuals [5, 14, 22–26]. The antigenicity of thyroid autoantigens (thyroglobulin and TPO) is increased after iodine exposition. The iodine is able to increase the amount of free radicals which are produced in the process of physiological hormonogenesis in the thyroid gland. In addition, there are accumulating data for antiviral capacity of Se. Both epidemiological and in vitro data demonstrated that Se deficiency might be important in viral infections as well [11]. Since the viruses have been published to induce IFN-*γ*, consequently HLA-DR expression, therefore, it is hypothesized that the trigger in autoimmune thyroiditis might be a virus infection [27–29]. Nowadays, the suggestion of viral origin of autoimmunity appears to be a speculation; however, the “selenium story” might open a new window not only for better understanding of beneficial effect of Se in autoimmune thyroiditis but also in the research of origin of autoimmunity [9, 11–13, 15, 22]. A new perspective has been opened by the investigations of Se on the role of T regulatory cells (Treg) with CD4CD25 FoxP3 markers [24, 25, 30, 31]. Accumulating data demonstrated that deficiency of CD4^+^CD25^+^ Treg cells was closely correlated with development of ATD [24, 25, 30, 31]. Recently, it was published in animal experiments that the CD4CD25 FoxP3 T cells displayed preventive effect on development of ATD [26]. Surprisingly, Se upregulated CD4^+^CD25^+^ regulatory T cells in iodine-induced autoimmune thyroiditis model of NOD.H-2^h4^ mice [24]. Our observation and experiments provide evidence that Se has a complex effect on immune system including decreased expression on HLA-DR molecules and by this way can prevent the induction and perpetuation of autoimmune thyroid processes.

## 5. Conclusions

Se has a dose-dependent inhibitory effect on the expression of HLA-DR molecules of thyrocytes induced by interferon-*γ*. This effect of selenium was in the inverse correlation with anti-oxidative capacity. Inhibitory effect of Se on HLA-DR molecule expression and antioxidative capacity is involved into therapy of autoimmune thyroiditis. Our in vitro study provided evidence that the free radical scavenging effect of Se plays an important role in the therapy and the prevention of autoimmunity.

## Figures and Tables

**Table 1:** Investigation of HLA-DR expression on thyrocytes induced by IFN-*γ* in the absence and presence of selenium. Figures in the table represent mean percentage DR positive human thyroid cells ±SD. All experiments were made in triplicate. ns. = not significant.

Culture of human thyrocytes	Expression of HLA-DR on thyrocytes (%)				
Culture media (*n* = 4)	3.7 ± 2.4	*P* < 0.001			
IFN-*γ* (100 U/mL)	35.2 ± 5.2		n.s	*P* < 0.05	*P* < 0.001
IFN-*γ* (100 U/mL) + sodium selenite (10 nM/mL) (*n* = 3)	33.2 ± 14.7				
IFN-*γ* (100 U/mL) + sodium selenite (50 nM/mL) (*n* = 3)	26.4 ± 12.7				
IFN-*γ* (100 U/mL) + sodium selenite (100 nM/mL) (*n* = 3)	11.5 ± 5.2				

## Abbreviations

AIT: Autoimmune thyroiditis
ATD: Autoimmune thyroid disease
HLA: Human leucocyte antigen DR locus
IFN-*γ*: Interferon-*γ*
MHC: Major histocompatibility complex
Se: Selenium
TAS: Total antioxidant state
TPO: Thyroid peroxidase
APCs: Antigen presenting cells.
